# π-Helix controls activity of oxygen-sensing diguanylate cyclases

**DOI:** 10.1042/BSR20193602

**Published:** 2020-02-20

**Authors:** Johnnie A. Walker, Yuqi Wu, Jacob R. Potter, Emily E. Weinert

**Affiliations:** 1 Department of Chemistry, Emory University, 1515 Dickey Dr. NE, Atlanta, GA 30322, U.S.A.; 2 Department of Biochemistry and Molecular Biology, Pennsylvania State University, 306 Althouse Laboratory, University Park, PA 16802, U.S.A.

**Keywords:** diguanylate cyclase, globin, heme, oxygen sensing, signaling protein

## Abstract

The ability of organisms to sense and adapt to oxygen levels in their environment leads to changes in cellular phenotypes, including biofilm formation and virulence. Globin coupled sensors (GCSs) are a family of heme proteins that regulate diverse functions in response to O_2_ levels, including modulating synthesis of cyclic dimeric guanosine monophosphate (c-di-GMP), a bacterial second messenger that regulates biofilm formation. While GCS proteins have been demonstrated to regulate O_2_-dependent pathways, the mechanism by which the O_2_ binding event is transmitted from the globin domain to the cyclase domain is unknown. Using chemical cross-linking and subsequent liquid chromatography-tandem mass spectrometry, diguanylate cyclase (DGC)-containing GCS proteins from *Bordetella pertussis* (*Bpe*GReg) and *Pectobacterium carotovorum* (*Pcc*GCS) have been demonstrated to form direct interactions between the globin domain and a middle domain π-helix. Additionally, mutation of the π-helix caused major changes in oligomerization and loss of DGC activity. Furthermore, results from assays with isolated globin and DGC domains found that DGC activity is affected by the cognate globin domain, indicating unique interactions between output domain and cognate globin sensor. Based on these studies a compact GCS structure, which depends on the middle domain π-helix for orienting the three domains, is needed for DGC activity and allows for direct sensor domain interactions with both middle and output domains to transmit the O_2_ binding signal. The insights from the present study improve our understanding of DGC regulation and provide insight into GCS signaling that may lead to the ability to rationally control O_2_-dependent GCS activity.

## Introduction

Since the discovery of cyclic dimeric guanosine monophosphate (c-di-GMP) as a bacterial second messenger and its effects on biofilm formation and antibiotic resistance, the proteins involved in c-di-GMP pathways have been of considerable interest as possible targets for the development of new antibiotics [1–4]. To date, conserved motifs for the enzymes responsible for c-di-GMP production (diguanylate cyclase; GGDEF) and c-di-GMP hydrolysis (c-di-GMP phosphodiesterase; EAL or HDGYP) have been identified, allowing for relatively facile identification of bacteria that utilize c-di-GMP signaling to control cellular phenotypes [5,6]. However, our understanding of how c-di-GMP metabolic enzymes are regulated and how bacteria obtain disparate downstream effects from activation of different diguanylate cyclases is incomplete. In particular, the molecular mechanisms by which environmental signals regulate c-di-GMP production remain poorly understood, despite the importance of this information for both understanding signal-specific c-di-GMP signaling and designing anti-biofilm agents [6–8].

Oxygen (O_2_) levels have been demonstrated to regulate biofilm formation and to affect virulence, making it an important external signal, likely due to its importance in metabolism [9–11]. Globin coupled sensor (GCS) proteins utilize a heme-containing globin domain to sense O_2_ levels and transmit the ligand-binding signal through a linking middle domain to an output domain. GCS proteins have been identified in numerous bacteria with a variety of output domains that are regulated by the O_2_-binding signal, including methyl accepting chemotaxis protein (MCP), kinase, and diguanylate cyclase (DGC) [12,13]. In *Bacillus subtilis*, the MCP-containing GCS regulates aerotaxis, allowing the organism to move to its preferred location within an O_2_ gradient [14]. In contrast, the DGC-containing GCS from the plant pathogen *Pectobacterium carotovorum* (*Pcc*GCS) regulates O_2_-dependent motility, virulence factor production, and rotting within a plant host, highlighting the importance of O_2_ sensing and GCS proteins in controlling bacterial phenotypes [11].

Within the GCS protein family, the most common effect of O_2_ binding to the heme of the globin domain is an increase in the activity of the output domain [13]. This increase in GCS enzymatic activity often requires oligomerization of GCS monomers to yield activity of the output domains; GCS proteins with diguanylate cyclase, histidine kinase, and adenylate cylcase output domains have been shown to function as homodimers and higher order oligomers [15–20]. Because of the multi-domain organization and oligomerization of GCS proteins, questions have arisen regarding the overall structure and path of signal transduction within the protein family. A number of studies have focused on elucidating the structure of GCS proteins, but to date, high resolution structural information is only available for isolated domains. Sensor globin domains from HemAT-*Bs* (MCP-GCS from *B. subtilis*), *Af*GcHK (kinase-GCS from *Anaeromyxobacter* sp. FW109-5), *Ec*DosC (DGC-GCS from *E. coli*), and *Bpe*GReg (DGC-GCS from *Bordetella pertussis*) [21] have been crystallized as dimers in different ligation states (Fe^II^, Fe^II^-CO, Fe^III^-CN, Fe^II^-O_2_, Fe^III^-H_2_O) and have highlighted some conformational changes within the globin domain that might be involved in signal transduction [22–24]. These include displacement of the globin monomers relative to each other, changes in helix flexibility (as evidenced by B factors), and subtle changes in heme pocket conformation. Structures of the isolated middle and DGC domains from *Ec*DosC have provided additional insights [24]. The middle domain was demonstrated to adopt a four-helix bundle architecture containing a short π-helix and to form a dimer in the crystal structure, suggesting a possible dimeric configuration within the full-length protein. In addition, one crystal form of the DGC domain formed a dimer with the product inhibitory sites (I-sites) near the dimer interface, highlighting a potential mechanism by which the DGC domain could exert the product inhibition observed in enzymatic assays.

Despite the insights gained from individual domain structures, these structures have not provided detailed information regarding transfer of the O_2_-binding signal from the globin domain to the output domain. In a study of *Af*GcHK (kinase-containing GCS), which is dimeric, hydrogen–deuterium exchange mass spectrometry (HDX-MS) was used to determine regions within the GCS that might undergo ligand-dependent conformational changes. The HDX-MS data indicated that the distal side of the heme pocket of the globin domain, the region of the peptide backbone that interacts with gaseous ligands, was very flexible and thus probably involved in signal transmission by globin conformational changes [17]. However, investigations of full-length *Ec*DosC demonstrated that it exists as a homodimer with a somewhat elongated shape and, based on charge complementarity analysis, was proposed to adopt a conformation with the globin domains orientated at one end of the middle domain dimer through flexible linkers and the DGC dimer connected through small loops at the other end of the middle domain dimer. This proposed model would suggest that the distal heme pocket would have to propagate the ligand binding signal through rearrangements of the middle domain, as this model does not predict interaction between the globin and DGC domains [24].

In the present study, we have used alternative methods to gain insights into the signal transmission between domains of two previously studied DGC-containing GCSs, *Pcc*GCS from *Pectobacterium carotovorum* and *Bpe*GReg from *Bordetella pertussis* [15,25]. *Bpe*GReg and *Pcc*GCS have been chosen for analysis due to the previously reported ligand binding and enzyme kinetic characterizations [15,25], as well as the finding that *Pcc*GCS signaling controls O_2_-dependent soft rot infections of crop plants [11]. First, chemical cross-linking followed by protease digestion and liquid chromatography-tandem mass spectrometry (LC-MS/MS) analysis was used to identify interactions between domains within the two DGC-containing GCS proteins. Next, influenced by the cross-linking results and previous *Bpe*GReg mutation studies by Wan and others [9,10], conserved histidine and lysine residues within a π-helical region of the middle domain were mutated in *Pcc*GCS, and this mutant was compared biochemically and biophysically to the wild-type form to show that the π-helix mutations do affect DGC activity. Furthermore, enzyme kinetic assays utilizing isolated diguanylate cyclase and globin domains of *Pcc*GCS revealed enhanced activity due to specific interactions of the cognate globin domain. Finally, the globin domain from *Bpe*GReg was used to probe the specificity of globin–DGC interactions. The results reported herein suggest an approximate quaternary structure that is needed for these DGC GCSs to function. The middle domain π-helix appears to be required for the proper quaternary structure formation and, based on *Pcc*GCS, the globin domain makes contacts with the DGC domain that can affect DGC activity. The present study provides an improved understanding of the structure of DGC GCSs and the mechanism of output domain regulation within DGC GCSs.

## Materials and methods

### Gene cloning

Full-length *Pcc*GCS and *Bpe*GReg and *Pcc*Globin and *Bpe*Globin constructs were cloned previously into vector pET20b [25,26]. *Pcc*GCS H237A/K238A was created through site directed mutagenesis by PCR. Site-directed mutagenesis primers were 5′-TGGTTCAACGCGGCGGGTAAGTCATCGTTCAGCAATATC-3′ (Forward) and 5′-TGACTTACCCGCCGCGTTGAACCACAGGCCAAATTCGGA-3′ (Reverse). The MBP-*Pcc*DGC construct was created using the vector pMAL-c2x (New England Biolabs, Ipswich, MA). A his-tag (5′-ATGCATCATCATCATCATCATCACCTGAAAATCGAAGAAGGTCATCATCATCATCATCACCTG-3′) replaced the original start codon, and a tobacco etch virus (TEV) protease cleavage site (ENLYFQS (5′-GAGAACCTGTACTTCCAGTCA-3′)) was inserted between the Factor Xa cleavage site and *Eco*RI restriction site to create vector pHis-MBP. Using polymerase incomplete primer extension (PIPE) cloning [27], an *Escherichia coli* codon-optimized DNA sequence for residues #321 – 470 (N- LPTILRHE….EYAQEE -C) of *Pcc*GCS (DGC domain) was inserted into the multiple cloning site using four primers to create pHis-MBP-*Pcc*DGC. The primers used to amplify pHis-MBP were 5′-CAGTCAGAATTCGGATCCTCTGGCCTGCCGACCATCCTGCGCCATGAA-3′ (Forward) and 5′-CGTTGTAAAACGACGGCCAGTGCCAAGCTTTTATTCTTCTTGAGCGTATTCAAT-3′ (Reverse). The primers used to amplify the DGC domain from pET20b-*Pcc*GCS were 5′-CTGCCGACCATCCTGCGCCATGAAATTTCACTGGCG-3′ (Forward) and 5′-GATGGTCGGCAGGCGACCTTCGATGTGGTGGTGGTG-3′ (Reverse).

### Protein expression and purification

The expressions and purifications of full-length *Pcc*GCS (WT and H237A/K238A) and *Bpe*GReg and *Pcc*Globin and *Bpe*Globin constructs were conducted as previously described [25,26]. Plasmid pHis-MBP-*Pcc*DGC was transformed into *E. coli* strain C41(DE3) pLysS (Lucigen, Middleton, WI) and MBP-*Pcc*DGC expression was carried out by inducing cells at OD_600_ ∼ 0.6–0.8 with 100 μM IPTG for ∼20 h at 20°C with expression media containing 45 g/l yeast extract, 1% glycerol (v/v), 1.6 g KH_2_PO_4_ and 6.27 g K_2_HPO_4_ per liter. Buffer A (50 mM Tris pH 7.0, 300 mM NaCl, 5% glycerol (v/v), 20 mM imidazole) was used to resuspend MBP-*Pcc*DGC cell pellets, and cell lysis was completed using a homogenizer at 4°C (Avestin, Inc., Ontario, Canada). After centrifugation at 130,000 × ***g*** at 4°C for 1 h (Beckman Optima L-90X ultracentrifuge), the supernatant was loaded onto a Buffer A pre-equilibrated column containing HisPur Ni resin (Fisher Scientific, Hampton, NH). Bound protein was eluted with Buffer B (50 mM Tris pH 7.0, 300 mM NaCl, 5% glycerol (v/v), 250 mM imidazole) and buffer-exchanged/desalted with Buffer C (50 mM Tris pH 7.0, 50 mM NaCl, 5% glycerol (v/v), 1 mM DTT) on a S200 gel filtration column (GE Healthcare, Chicago, IL). The collected protein was concentrated (Vivaspin 10 kDa MWCO centrifugal concentrators; Sartorius, Goettingen, Germany), flash frozen in liquid N_2_, and stored at −80°C. Protein identity/purity was assessed by SDS-PAGE.

### Cross-linking and liquid chromatography-mass spectrometry/mass spectrometry

Cross-linking was accomplished by incubating 20 μM of *Pcc*GCS/*Bpe*GReg with 2.5× (50 μM), 5× (100 μM) and 20× (400 μM) of bis(sulfosuccinimidyl) suberate-d_0_ (BS3-d_0_) for ∼35 min at room temperature, and the reactions were stopped by adding ammonium bicarbonate to a final concentration of 20 mM. The reactions then were run on Bio-Rad 7.5% Criterion gels to separate oligomers for gel removal (Supplementary Figure S2).

The bands representing different oligomers were excised, and 50% acetonitrile in 50 mM ammonium bicarbonate was used to destain the gel pieces. The samples were dehydrated using 100% acetonitrile and by vacuum. Trypsin digestion was completed by the addition of 10 ng/μl trypsin in 50 mM ammonium bicarbonate to the dried gel pieces and incubation overnight at 37°C. Peptides were extracted using an extraction buffer consisting of 50% acetonitrile in 5% formic acid, and the extracted peptides were dehydrated with 100% acetonitrile and by vacuum. The final cleanup step involved reconstitution with 0.1% trifluoroacetic acid, desalting with a C18 microcolumn [28], and drying under vacuum.

The prepared peptides were resuspended in loading buffer (0.1% formic acid, 0.03% trifluoroacetic acid, 1% acetonitrile) and separated on a C18 (1.9 μm, Dr Maisch, Germany) fused silica column (15 cm × 75 μM internal diameter (ID); New Objective, Woburn, MA) by a Dionex Ultimate 3000 RSLCNano and monitored with a Fusion mass spectrometer (ThermoFisher Scientific, San Jose, CA). Elution of peptides was achieved by a gradient with Buffer E ranging from 3 to 65% with a mobile phase of Buffer D before gradient (Buffer D – 0.1% formic acid in water, Buffer E – 0.1% formic acid in acetonitrile). Collection at the top speed for 3 s cycles was programmed for the mass spectrometer cycle. Electron-transfer dissociation fragmentation for all precursor ions with charges of 3–8 and higher energy collision dissociation fragmentation for ions with charges of 2–5 were performed by a decision tree. A resolution of 120,000 at *m/z* 200 in profile mode was used in mass spectrometry scans (400–1600 *m/z* range, 200,000 AGC, 50 ms maximum ion time), and electron-transfer dissociation (0.7 *m/z* isolation width, charge dependent collision energy, 10,000 AGC target, 35 ms maximum ion time) and high-energy collision (0.7 *m/z* isolation width, 32% collision energy, 10,000 AGC target, 35 ms maximum ion time) MS/MS spectra were detected in the ion trap. Dynamic exclusion omitted previously sequenced precursor ions for 20 s within a 10 ppm window, and precursor ions with +1 and +9 or higher charge states were removed from sequencing.

Data analysis was performed using Spectrum Identification Machine (SIM-XL) [29,30]. SIM-XL uses a unique paradigm for search-space reduction and utilizes reporter ions for identifying tandem mass spectra derived from cross-linked peptides. Settings for data analysis were: crosslinker – disuccinimidyl suberate DSS; mass shift – 138.0681; modification mass shift – 156.0786; reaction sites – KK,KS,KN-Term; reporter ions – 222.149, 239.1759, 240.159, 305.2229; precursor ppm – 20; fragment ppm – 20; Xrea threshold – 0; enzyme specificity – fully specific; enzyme – trypsin; enzyme regular expression – [KR](?!P); enzyme C/N terminal – C-terminal; deconvoluted MS/MS – false; fragmentation method – HCD; number of isotopic possibilities – 4; minimum amino acid residues per chain – 4; intra-link max charge – 4; maximum missed cleavages – 3; peaks matched cutoff – 0; minimum MH (linear peptide) – 600; maximum MH (linear peptides) – 6000. Identified cross-links were screened manually for cross-links with inter-link and intra-link scores of 2.0 or greater and fragmentation spectra peaks representing y (C-terminal fragment) and b (N-terminal fragment) ions that include the crosslinked residues as suggested by the developers of SIM-XL [30] (Supplementary Figures S5 and S6).

### Enzyme kinetics

Enzyme kinetics assays were performed with the EnzChek pyrophosphate kit (ThermoFisher Scientific, Waltham, MA), as previously described [15,25]. Proteins were reduced and the ligation/oxidation states were confirmed by UV–Vis spectroscopy before the assays were performed. Enzyme kinetics with Fe^II^-unligated globin and maltose binding protein (MBP) linked-*Pcc*DGC were performed in an anaerobic chamber in the presence of ∼75 μM sodium dithionite, while an anaerobically prepared EnzChek kit was used for Fe^II^-unligated *Pcc*GCS WT and H237A/K238A reactions. EnzCheck kit instructions were followed and reactions were initiated with 500 μM GTP. Assays were performed in 96-well plates and in triplicate. Three concentrations each of *Pcc*GCS WT and H237A/K238A with *Ec*DosP (a c-di-GMP phosphodiesterase used at three times the amount of *Pcc*GCS to prevent DGC product inhibition) were used to determine turnover numbers in *V*_max_ conditions. In the globin titration experiments, MBP-*Pcc*DGC concentration in all reactions was 3 μM, and *Ec*DosP was used at the same concentration. *Pcc*Globin was added at 10× (30 μM) and 20× (60 μM) concentrations, while *Bpe*Globin was added at 20× (60 μM) to probe for globin-induced effects. Reactions were monitored for 4 h using Epoch plate readers with Gen5 software (Biotek Instruments, Inc., Winooski, VT), and data analysis was performed using Igor Pro (Wavemetrics, Portland, OR).

### Circular dichroism

Spectra for both Fe^II^-O_2_ *Pcc*GCS WT and H237A/K238A were recorded with a JASCO J-1500 CD spectrometer (JASCO, Easton, MD) that was chilled by liquid nitrogen. Proteins were reduced within an anaerobic chamber using sodium dithionite, buffer exchanged into Buffer E (100 mM sodium phosphate pH 7.0), and then mixed with oxygenated Buffer E. Concentrations of proteins were ∼50 μM. Spectra shown are averages of three scans of the protein samples at 20°C.

### Analytical gel filtration

Size-exclusion chromatography using an Agilent 1200 infinity system with a Sepax SEC-300 (7.8 mm Å × 300 mm, 300 Å), and diode array detector (simultaneous detection at 214, 416 and 431 nm) was used to detect the oligomers of *Pcc*GCS WT and H237A/K238A. Sample preparation occurred as follows: proteins reduced in an anaerobic chamber, proteins buffered exchanged into Buffer D (50 mM Tris pH 7.0, 50 mM NaCl, 1 mM DTT), and proteins mixed with oxygenated Buffer D. The mobile phase was 150 mM sodium phosphate pH 7.0 for all experiments. Calibration curves were determined using ferritin (443 kDa), β-amylase (200 kDa), alcohol dehydrogenase (150 kDa), bovine serum albumin (66 kDa) and carbonic anhydrase (29 kDa) as globular protein molecular weight standards (MilliporeSigma, St. Louis, MO).

### Electronic absorption spectroscopy

Spectra for *Pcc*GCS H237A/K238A were recorded with an Agilent Cary 100 with Peltier accessory (Agilent Technologies, Santa Clara, CA). Sample preparation followed protocols previously described except protein samples were prepared in Buffer D [25,26,31,32].

### Stopped-flow ultraviolet–visible spectroscopy

Determination of O_2_ dissociation rates was completed following previously described procedures [15,25,26]. *Pcc*GCS H237A/K238A was reduced in an anaerobic chamber using sodium dithionite, buffered exchanged into Buffer D, removed from the anaerobic chamber, and then mixed with oxygenated Buffer D. Rapid mixing with an equal volume of sodium dithionite solution (50 mM Tris pH 7.0, 50 mM NaCl, 1 mM DTT, 5 mM sodium dithionite) occurred within a SX20 stopped-flow apparatus (Applied Photophysics). O_2_ dissociation was monitored with a diode array detector. Global fitting was completed with Pro-KII software (Applied Photophysics) and additional fitting analysis handled with Igor Pro (Wavemetrics, Portland, OR).

### Isothermal titration calorimetry

Substrate affinity was determined using a Microcal Auto-iTC200 within the Pennsylvania State University Huck Institute Automated Biological Calorimetry Core facility. *Pcc*GCS H237A/K238A was buffer exchanged into 100 mM potassium phosphate, pH 7.0, and used at a concentration of 80 μM. GTP and two non-hydrolyzable substrate analogues (guanosine-5′-[(α,β)-methyleno]triphosphate, sodium salt (GTP-α-S), and guanosine-5′-(α-thio)-triphosphate, sodium salt (GpCpp); Jena Biosciences) were dissolved in 100 mM potassium phosphate, pH 7.0, buffer to a stock concentration of 600 μM. The cell was maintained at 25°C, stirred at 750 rpm, and contained 200 μl of protein. Twenty injections of 2 μl of GTP/analog were made over ∼1.5 h.

## Results

### Cross-linking of *Pcc*GCS and *Bpe*GReg

**Figure 1:**
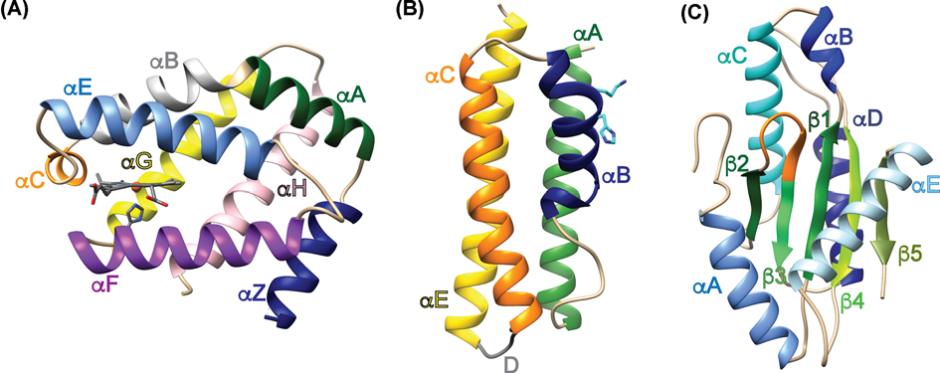
Homology models Homology models of the globin, middle, and diguanylate cyclase domains of *Pcc*GCS. (**A**) Globin domain (heme represented by stick-and-ball structure). (**B**) Middle domain showing the conserved histidine-237 and lysine-238 in cyan. (**C**) Diguanylate cyclase domain (GGEEF active site residues are shown in orange). Homology models were made for both *Pcc*GCS and *Bpe*GReg using *Ec*DosC crystal structures and the Protein Homology/analogY Recognition Engine V2.0 (PHYRE 2) tool [35]. Structure molecular graphics were created using the UCSF Chimera package [39].

**Figure 2:**
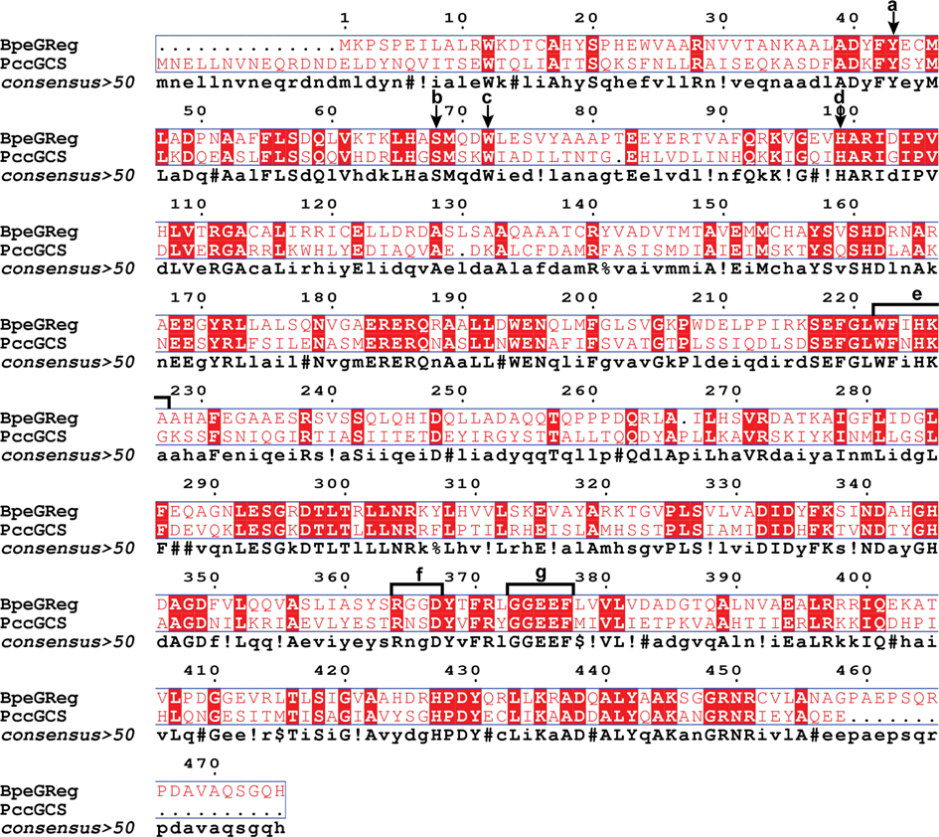
Sequence alignments Sequence alignment of *Bpe*GReg and *Pcc*GCS. (**A**) Conserved distal tyrosine (hydrogen bonds with oxygen). (**B**) Conserved distal serine (helps stabilize bound oxygen). (**C**) Conserved tryptophan (in contact with edge of heme). (**D**) Conserved proximal histidine (holds heme in globin domain). (**E**) Conserved π-helical region. (**F**) Product-binding inhibitory site (I-site) motif (RxxD). (**G**) Active site motif (GGEEF). Alignment and image created using MultAlin [40] and ESPript 3.0 [41].

**Figure 3:**
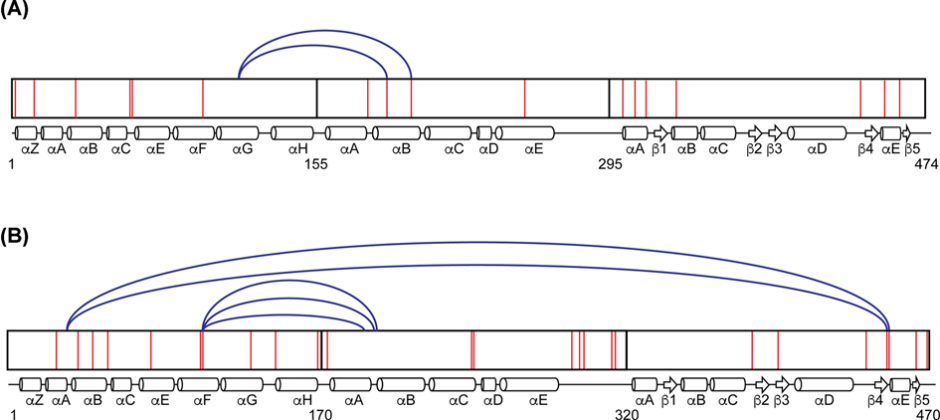
Cross-linking analysis Cross-linking maps of *Bpe*GReg and *Pcc*GCS tetramer forms. (**A**) Cross-links for *Bpe*GReg. (**B**) Cross-links for *Pcc*GCS. The three domains of the proteins are shown with the arrangement of their secondary structures (drawn approximately to scale). Lysine residues are shown as red lines within the domains and numbering is based on native residue numbering. Cross-links were identified using Spectrum Identification Machine [29,30].

Cross-linking and tandem mass spectrometry/mass spectrometry were utilized in an effort to determine if there are unique interactions between the three GCS domains. *Pcc*GCS WT (Uniprot accession number: C6D9C2) consists of four domains, based on sequence alignments and domain homology, with the following approximate boundaries: HisTag (residues 1–11), globin domain (residues 12–181), middle domain (residues 182–331) and DGC domain (residues 332–481) (Figures 1 and 2). Structural features are labeled based on homology models generated using structures of the individual domains of *Ec*DosC (Figure 1). To identify domain interactions, *Pcc*GCS WT was cross-linked with an electrophilic crosslinker, different oligomeric states separated using SDS-PAGE, and then each oligomer subjected to tryptic digest/LC-MS/MS analysis to identify cross-linked peptides. *Pcc*GCS WT contains 25 lysine residues throughout the 482 amino acid sequence, which are the preferred sites for succinimide cross-linkers. After screening of numerous cross-links by examining the spectra, five crosslinks were found for the tetramer form of *Pcc*GCS WT (Figure 3; Supplementary Table S1); three globin domain (helix αF)-middle domain (helix αB) and two globin domain (helix αB)-DGC domain (helix αD) cross-links identified. The dimer form of *Pcc*GCS WT did not produce cross-links with spectra that passed screening.

In analogy to *Pcc*GCS, the *Bpe*GReg WT construct (Uniprot accession number: Q7VTL8) consists of four segments (HisTag (residues 1–11), globin domain (residues 12–166), middle domain (residues 167–306), and DGC domain (residues 307–485); Figure 2), and cross-linking for *Bpe*GReg was accomplished in the same manner as described above for *Pcc*GCS. *Bpe*GReg contains 17 lysine residues throughout the 485 amino acid sequence (Figure 2). There were only two globin domain (loop between helices αG and αH)-middle domain (helix αB) cross-links identified after inspecting the cross-linking spectra for the tetramer form of *Bpe*GReg (Figure 3). No cross-linking spectra for the dimer form of *Bpe*GReg passed screening, which followed results from *Pcc*GCS WT in dimer form. These seven cross-links from *Pcc*GCS and *Bpe*GReg provide evidence for globin domain interactions with the middle and output domains.

As the different oligomeric states of *Pcc*GCS and *Bpe*GReg were isolated prior to MS analysis, at least one identified cross-link must be between monomers within the tetrameric complexes. However, due to the challenges with generating tetrameric assemblies consisting of four labeling patterns, we were unable to determine which cross-links correspond to inter-monomer cross-links and which are intra-monomer linkages.

### Effect of π-helix disruption on diguanylate cyclase activity

**Figure 4:**
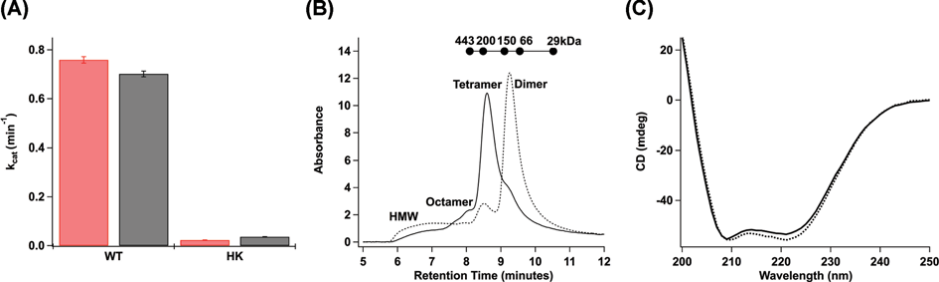
Biochemical effects of π-helix mutations Effects of π-helix mutations on *Pcc*GCS characteristics. (**A**) Enzyme kinetics of *Pcc*GCS WT (WT) and H237A/K238A (HK). Red values represent proteins in the ligated Fe(II)-O_2_ state, and black values represent proteins in the unligated Fe(II) state. Turnover numbers were calculated from assays at *V*_max_ conditions with varying enzyme concentrations, and error bars represent standard deviations. (**B**) Analytical gel filtration analysis of oligomerization states of *Pcc*GCS WT (solid line) and H237A/K238A (dotted line). Different oligomeric states are annotated for both constructs on the graph. The molecular weights and retention times of globular protein standards are plotted above the graph. (**C**) Circular dichroism spectra for *Pcc*GCS WT (solid line) and H237A/K238A (dotted line). The plots are the averages of three scans for each construct.

The globin-coupled sensor middle domain contains consecutive conserved histidine and lysine amino acid residues, and these *Pcc*GCS residues were mutated to alanines (native residues numbering – H237A and K238A) to disrupt the π-helical nature of helix αB (refer to Figures 1B and 2). Previous work with *Bpe*GReg mutated conserved histidine-225 to alanine (native residues numbering); this mutation led to a construct with undetectable c-di-GMP production from GTP [9]. The production of c-di-GMP from GTP in the presence of phosphodiesterase (to remove product inhibition) was measured for both WT and H237A/K238A variants of *Pcc*GCS. The turnover numbers of *Pcc*GCS H237A/K238A in aerobic and anaerobic conditions were ∼30× (2.27 × 10^−2^ min^−1^) and ∼20× (3.62 × 10^−2^ min^−1^), respectively (∼1.5-fold increase in catalysis upon O_2_ binding), less than the aerobic and anaerobic turnover numbers of *Pcc*GCS WT (7.59 × 10^−1^ min^−1^ and 7.01 × 10^−1^ min^−1^, respectively; ∼2.5-fold increase in catalysis upon O_2_ binding) (Figure 4A). Isothermal titration calorimetry (ITC) was used to determine if the decreased enzyme activity of *Pcc*GCS H237A/K238A was due to changes in the rate of catalysis, substrate affinity, or a combination thereof. Data from titration with GTP was not useable due to the heat generated from diguanylate cyclase catalysis, even with the very slow rate. Binding of the non-hydrolyzable analog GTP-α-S was very weak (*K*_d_ > 300 μM) and therefore were not pursued further. In contrast, titration of *Pcc*GCS H237A/K238A with GpCpp yielded a *K*_d_ of 9.7 ± 3.9 μM (Supplementary Figure S7). For comparison, the *Pcc*GCS WT *K*_M_ for GTP was previously measured to range from 31 to 62 μM, depending on heme ligation state [25]. Therefore, disruption of the π-helix within the middle domain results in a drastic loss of *Pcc*GCS diguanylate cyclase activity due to decreased enzymatic catalysis, not substrate binding.

### Biophysical characterization of *Pcc*GCS π-helix mutant

The structures of the *Pcc*GCS WT and H237A/K238A variants were explored to determine if structural changes caused the loss of DGC activity of the mutant. Circular dichroism was employed to investigate the secondary structure compositions of both *Pcc*GCS variants, and no significant differences were seen within the spectra of the two variants (Figure 4C). *Pcc*GCS oligomerization was observed using analytical gel filtration to ascertain if there were tertiary structural changes that would help explain the changes in DGC activity. The dominant oligomeric state shifted from tetramer (WT) to dimer (H237A/K238A), a significant change in quaternary structure, and possibly tertiary structure, due to the π-helix-disrupting mutations (Figure 4B).

No change in ligand-binding by the globin domain was seen with disruption of the π-helix. The absorption spectra of *Pcc*GCS H237A/K238A in various ligation states are identical with those of *Pcc*GCS WT (Supplementary Figure S3), and the mutant displayed biphasic O_2_ dissociation rates very similar to *Pcc*GCS WT with slow (*k*_1_) and fast (*k*_2_) rates of 0.68 ± 0.10 and 3.88 ± 0.32 s^−1^, respectively [25].

### Effect of Isolated globin domains on diguanylate cyclase activity

To directly probe interactions between GCS protein globin and output domains, the diguanylate cyclase domain of *Pcc*GCS (native residues numbering 321–470; Figure 2) was expressed as a N-terminal maltose-binding protein (MBP) fusion, which resulted in a soluble DGC construct termed MBP-*Pcc*DGC. All other non-fusion constructs that were tested resulted in insoluble protein. The globin domains from *Pcc*GCS (*Pcc*Globin; native residues numbering 1–176), and *Bpe*GReg (*Bpe*Globin; native residues numbering 1–161) [26] were used to further probe interactions between the globin and cyclase domains, and to determine if direct linkage through the middle domain is required for the globin domain to affect catalytic activity of the cyclase domain (Supplementary Figure S1).

**Figure 5:**
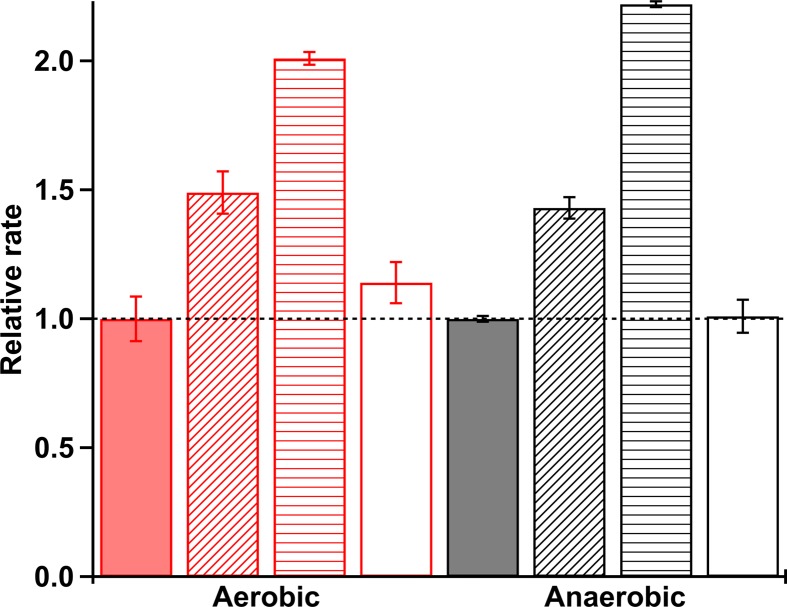
Diguanylate cyclase activity Isolated diguanylate cyclase activity in the presence of isolated globin domains. All rates are reported relative to *Pcc*DGC alone. Red bars, aerobic kinetics (Fe(II)-O_2_ globin); black bars, anaerobic kinetics (Fe(II) globin). Solid bars, *Pcc*DGC alone; diagonal stripes, *Pcc*DGC + 10× *Pcc*Globin; horizontal stripes, *Pcc*DGC + 20× *Pcc*Globin; empty bars, *Pcc*DGC + 20× *Bpe*Globin.

The catalytic rates of c-di-GMP production from MBP-*Pcc*DGC alone and in the presence of *Pcc*Globin or *Bpe*Globin were ascertained in the presence and absence of oxygen (Figure 5 and Supplementary S8). The aerobic rates of MBP-*Pcc*DGC supplied with 10× and 20× molar amounts of *Pcc*Globin were ∼1.5- and ∼2.0-fold, respectively, greater than the rate of c-di-GMP production for MBP-*Pcc*DGC alone (Figure 5). Similar results were seen for the anaerobic condition (Figure 5). However, the cyclase activity of MBP-*Pcc*DGC was not affected by the addition of 20× molar amount of *Bpe*Globin. These data demonstrate that the cognate globin domain is able to influence DGC activity through specific interactions, even in the absence of a direct linkage between the domains.

## Discussion

Given the ubiquity of c-di-GMP-related proteins in bacteria, understanding how regulatory domains modulate diguanylate cyclase activity will both improve our understanding of signal transduction within ligand-sensing DGC proteins and potentially identify new ways to target DGCs to inhibit biofilm formation [3,4,6–8]. Structures have been solved for a handful of full-length regulatory domain-DGC proteins and show two general domain organizations: (1) regulatory domain separated from DGC domain by an extended linker (such as WspR and PadC) and (2) regulatory domain and DGC domain in direct contact due to a very short/absent linking domain (including PleD and DgcZ) [16,33,34]. In the first case, ligand binding or phosphorylation of the regulatory domain leads to rearrangements that are propagated through the helical linking domain, leading to rotation of the DGC domains relative to each other and (in)activation. In contrast, activation of the regulatory domain in the second case results in rotation of the DGC domains to align the active site and allow for c-di-GMP production. However, to date, structural information is lacking for DGC proteins that contain substantial linking domains that do not form rod-like structures, such as GCS proteins.

Signal transduction following ligand binding to GCS proteins has been postulated to occur through conformational shifts in the globin domain that are propagated through the middle domain to reach the output domain [23,24]. The cross-linking and globin titration experiments described in this work demonstrate that GCS proteins adopt conformations that allow the globin domain to make interactions with both the middle and output domains of two DGC-containing GCS proteins, *Pcc*GCS and *Bpe*GReg (Figures 3 and 5). For *Bpe*GReg and *Pcc*GCS, both tetrameric forms exhibited multiple globin/middle cross-links. Two globin/DGC cross-links were observed in the tetramer form tested for *Pcc*GCS. To visualize the locations of residues with respect to one another, homology models of the three domains for *Pcc*GCS were created using the Protein Homology/analogY Recognition Engine V2.0 (PHYRE 2) tool (Figure 1) [35]. Taken together, these cross-links definitively demonstrate that GCS proteins can adopt conformations that allow both the globin and the middle domains to interact with the DGC output domain.

When comparing cross-linking of both proteins, the following region stood out: the αB helix/π-helical region of the middle domain (Figure 3). The existence of a π-helix within the middle domain of GCS proteins and the cross-links between the π-helix region and globin domain suggest a potential role in transmitting the O_2_-binding signal. π-Helices are defined by the occurrence of multiple hydrogen bonds between the amide backbone that reside in amino acids *i*+5 and *i* within an α-helix and require a bulge or insertion within a typical α-helix sequence. Mutation of residues within the bulge can alter the π-helix structure and function, but without significantly disrupting the α-helix [36]. A unique aspect of π-helices is the ability to move in peristaltic-like shifts that lead to structural changes within proteins [36]. Toluene 4-monooxygenase (T4moH), which consists of three polypeptides (TmoA, TmoE, and TmoB), is an enzyme that hydroxylates toluene with its cognate effector protein T4moD. In the toluene 4-monooxygenase/effector protein complex, active site changes occur due to a π-helical shift caused by T4moD binding to TmoH, which prepares the active site for substrate binding [37]. A π-helix also plays a role in infection caused by *Shigella* passing through the human small intestine. In response to high concentrations of the bile salt deoxycholate (DOC), which binds to invasion plasmid antigen D (IpaD) of the type three secretion system of *Shigella*, a π-helical shift within IpaD leads to association with invasion plasmid antigen B (IpaB) and eventually invasion of host cells [38]. Based on a sequence homology of middle domains from *Ec*DosC and 78 DGC-containing GCS homologues, the π-helical kink of *Ec*DosC (H223, K224) is highly conserved [24] and both these residues and the π-helical region are predicted in *Bpe*GReg (native residues numbering H225, K226) and *Pcc*GCS (native residues numbering H237, K238) (Figure 2).

For *Bpe*GReg, cross-links were observed between the globin αG/αH loop and the π-helical kink within helix αB of the middle domain. As the αG/αH loop in the globin was observed to become rigid after gas ligand binding to the heme within the histidine kinase-containing GCS from *Anaeromyxobacter* sp. Fw109-5 [23], this same increase in rigidity of the αG/αH helices loop may occur in *Bpe*GReg, which could result in signal transduction through a π-helical shift in the middle domain and increased DGC activity. Cross-links also show that *Pcc*GCS globin αF helix (contains proximal histidine that shifts due to gaseous ligand binding) interacts with the π-helical αB helix of the middle domain, suggesting that the middle domain π-helix is involved in signal transduction pathways in both *Bpe*GReg and *Pcc*GCS. The similar secondary structures (Figure 4) and ligand-binding abilities (Supplementary Figure S3) of the *Pcc*GCS WT and π-helix mutant (H237A/K238A construct) but major differences in DGC activities and oligomerization (Figure 4) suggest significant changes in quaternary structure and loss of π-helix interactions leading to interrupted signal transduction. In two previous studies by Wan and colleagues, mutation of the conserved histidine and lysine residues within *Ec*DosC and *Bpe*GReg led to inactive phenotypes for the two enzymes [9,10], supporting our data highlighting this region as being critical for enzyme activity of GCS proteins.

Interactions within *Pcc*GCS suggest protein structures wherein the domains are folded into a more compact structure than previously hypothesized for DGC-containing GCS proteins [24]. Cross-links were observed between the globin and cyclase domains within *Pcc*GCS, suggesting a mechanism of direct transduction of the ligand binding signal (Figure 3). The globin helix αB was linked to the helix αD of the DGC domain. Helix αB of the globin domain contains the distal tyrosine within the heme pocket that interacts directly with gaseous ligands. When a ligand binds to the globin heme, the signal could be transmitted to the DGC active site through the close association of the DGC helix αD with the active site αB-αC loop, which is involved in GTP binding (Figures 1–3).

To further probe the role of direct globin-DGC interactions, isolated *Pcc*Globin, *Bpe*Globin, and *Pcc*DGC domains were investigated. The addition of cognate globin domain, *Pcc*Globin, increased the activity of MBP-*Pcc*DGC in both aerobic and anaerobic conditions, whereas non-cognate *Bpe*Globin addition had no effect on MBP-*Pcc*DGC, even at 20-fold excess (Figure 5). These results demonstrate that *Pcc*Globin makes specific interactions with *Pcc*DGC that increase the rate of GTP to c-di-GMP conversion. However, without the middle domain linkage, *Pcc*Globin was no longer able to exert O_2_-dependent effects on diguanylate cyclase activity, suggesting that the middle domain is required to correctly position the globin domain relative to the DGC domain, as previous studies demonstrated higher catalytic activity for O_2_-bound full-length *Pcc*GCS (∼2.5-fold increase in cyclase activity for *Pcc*GCS WT Fe(II)-O_2_ vs. Fe(II)) [25].

A pairwise sequence alignment of the amino acid sequences of *Pcc*GCS and *Bpe*GReg was used to identify sequence characteristics that could result in differences in crosslinking patterns and globin-dependent MBP-*Pcc*DGC activity. The alignment indicates that the overall sequences are approximately 35% identical and 52% similar (Figure 2); *Pcc*GCS has a globin domain that is 12 amino acids longer (as an N-terminal extension) than the globin domain of *Bpe*GReg, while *Bpe*GReg has a larger DGC domain when compared to *Pcc*GCS (17 residue extension at the C-terminus). One possibility is a role for the 12-residue N-terminal extension of the PccGCS globin domain; however, cross-links were not observed between the globin extension and the DGC domain, possibly due to the absence of a lysine within the extension. The data did identify cross-links between the globin domain (helix αB) and the DGC (helix αD) of *Pcc*GCS, suggesting that interactions between the globin and DGC helices could be mediating the cognate interactions that allow for globin-specificity in activating MBP-*Pcc*DGC.

*Bpe*GReg and *Pcc*GCS cross-linking analysis, π-helix mutations, and the globin-DGC activity assays suggest that DGC-containing GCS proteins can form relatively compact structures, with the N-terminal globin domain directly interacting with the C-terminal diguanylate cyclase domain. These GCSs have a globular shape based on analytical gel filtration experiments [25], and the data presented here further support the formation of compact structures. Cross-linking data identified interactions between the DGC output domain and the sensor globin domain within *Pcc*GCS, suggesting that globin domain helices that undergo conformational changes due to ligand binding can directly transduce signal through interactions near the DGC active site. The middle domain αB helix/π-helical region has emerged as a central component of DGC-containing GCS proteins and appears to be crucial in maintaining the correct quaternary structure for function, even in the presence of direct globin-DGC domain interactions, as evidenced by the lack of O_2_-dependent changes in activity in the studies on isolated domains. Cross-linking interactions between the DGC output domain and the sensor globin domain within *Pcc*GCS, and the activity of the DGC domain of *Pcc*GCS being influenced by the cognate *Pcc*GCS globin domain, but not the *Bpe*GReg globin domain, are likely due to sequence differences. Based on the results of the present study, the proper quaternary structures of DGC GCSs that effectively convert GTP into c-di-GMP appear dependent on the middle domain/π-helical region for correct positioning of subunits within oligomers. Future structural studies will add to our understanding of how GCS structure leads to function and the mechanism of signal transduction within GCS proteins, and may allow for targeting GCS activity as a method to modulate bacterial biofilm formation and virulence.

## Supplementary Material

Supplementary Figures S1-S8 and Table S1

## Abbreviations

*Af*GcHK: GCS from *Anaeromyxobacter* sp. FW109-5
*Bpe*GReg: GCS from *Bordetella persussis*
c-di-GMP: cyclic dimeric guanosine monophosphate
DGC: diguanylate cyclase
*Ec*DosC: GCS from *Escherichia coli*
GCS: globin coupled sensor
GpCpp: guanosine-5′-[(α,β)-methyleno]triphosphate, sodium salt
GTP-α-S: guanosine-5′-(α-thio)-triphosphate, sodium salt
HDX-MS: hydrogen–deuterium exchange mass spectrometry
HemAT-*Bs*: GCS from *Bacillus subtilis*
ITC: isothermal titration calorimetry
LC-MS/MS: liquid chromatography-tandem mass spectrometry
MBP: maltose-binding protein
MCP: methyl accepting chemotaxis protein
*Pcc*GCS: GCS from *Pectobacterium carotovorum*
WT: wild-type
