# Supplementary material for: π-Helix controls activity of oxygen-sensing diguanylate cyclases
Source: Biosci Rep. 2020 Feb 20;40(2):BSR20193602. doi: 10.1042/BSR20193602 (PMC7033309; doi:10.1042/BSR20193602)
Supplement: Supplementary Figures S1-S8 and Table S1 [file BSR-2019-3602_supp.pdf]

Supplemental Data

π-Helix Controls Activity of Oxygen-Sensing Diguanylate Cyclases

Johnnie A. Walker, Yuqi Wu, Jacob R. Potter, and Emily E. Weinert

Table S1. Crosslinked peptides identified by SIM-XL analysis. Red indicates crosslinked residues.

***Bpe*GReg Tetramer.** Numbering based on native protein sequence.

AA1	AA2	Peptide 1	Peptide 2
129	216	RICELLDRDASLSAAQAAATCR	KSEFGLWFIHKAAHAFEGAAESR
129	226	RICELLDRDASLSAAQAAATCR	KSEFGLWFIHKAAHAFEGAAESR

***Pcc*GCS Tetramer.** Numbering based on native protein sequence.

AA1	AA2	Peptide 1	Peptide 2
45	411	AISEQK	LRKKIQDHPIHLQNGESITMTISAGIAVYSGHPDYECLIK
45	412	AISEQK	LRKKIQDHPIHLQNGESITMTISAGIAVYSGHPDYECLIK
107	213	WIADILTNTGEHLVDLINHQKKIGQIHAR	QNASLLNWENAFIFSVATGTPLSSIQDLSDSEFGLWFNHK
107	221	WIADILTNTGEHLVDLINHQKKIGQIHAR	QNASLLNWENAFIFSVATGTPLSSIQDLSDSEFGLWFNHK
107	222	WIADILTNTGEHLVDLINHQKKIGQIHAR	QNASLLNWENAFIFSVATGTPLSSIQDLSDSEFGLWFNHK

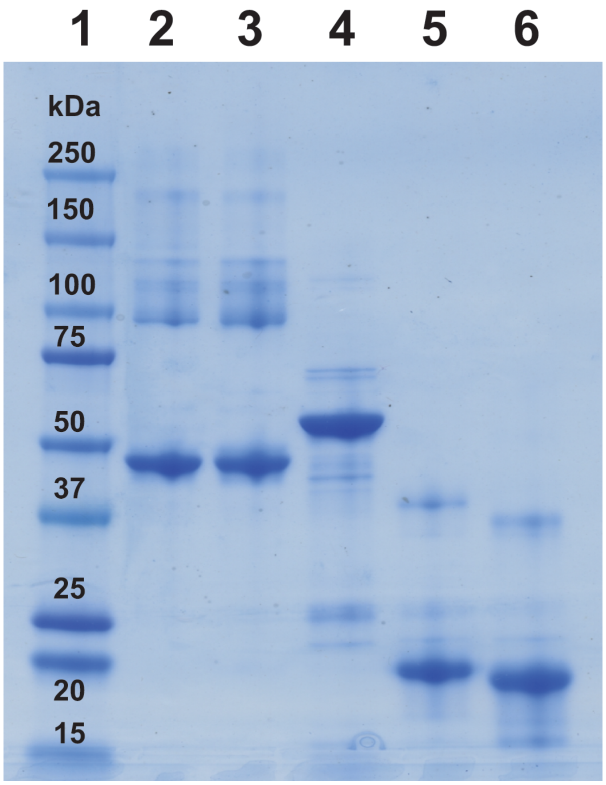

Figure S1. Purified protein constructs ran on a Bio-Rad 4-20% Criterion gel. Lane 1, Bio-Rad Precision Plus Kaleidoscope Protein Standards; Lane 2, *Pcc*GCS WT (55 kDa); lane 3, *Pcc*GCS H237A/K238A (55 kDa); lane 4, MBP-*Pcc*DGC (64 kDa); lane 5, *Pcc*Globin (22 kDa); lane 6, *Bpe*Globin (19 kDa).

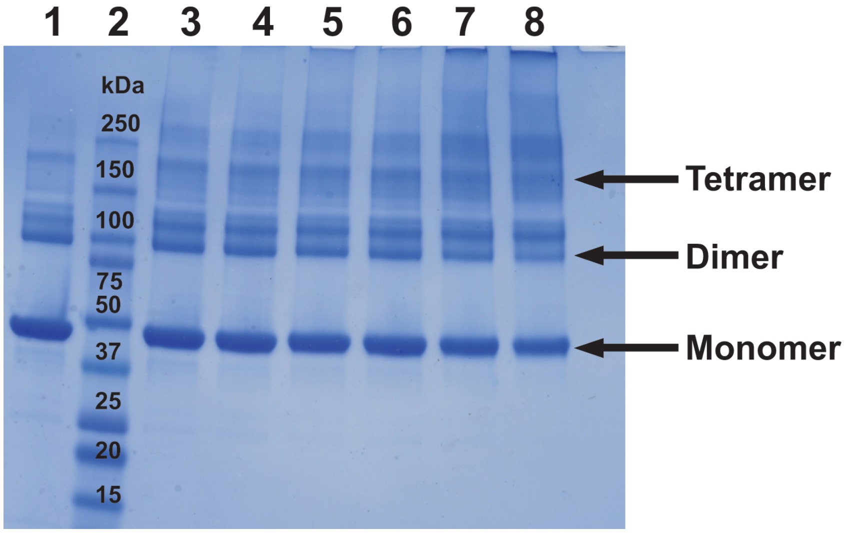

Figure S2. Example of a crosslinking polyacrylamide gel with *Pcc*GCS WT. Lane 1, *Pcc*GCS WT without crosslinker; lane 2, Bio-Rad Precision Plus Kaleidoscope Protein Standards; lanes 3 and 4, *Pcc*GCS WT with 2.5X BS3 crosslinker; lanes 5 and 6, *Pcc*GCS WT with 5X BS3 crosslinker; lanes 7 and 8, *Pcc*GCS WT with 20X BS3 crosslinker. This experiment was ran on a Bio-Rad 4-20% Criterion gel.

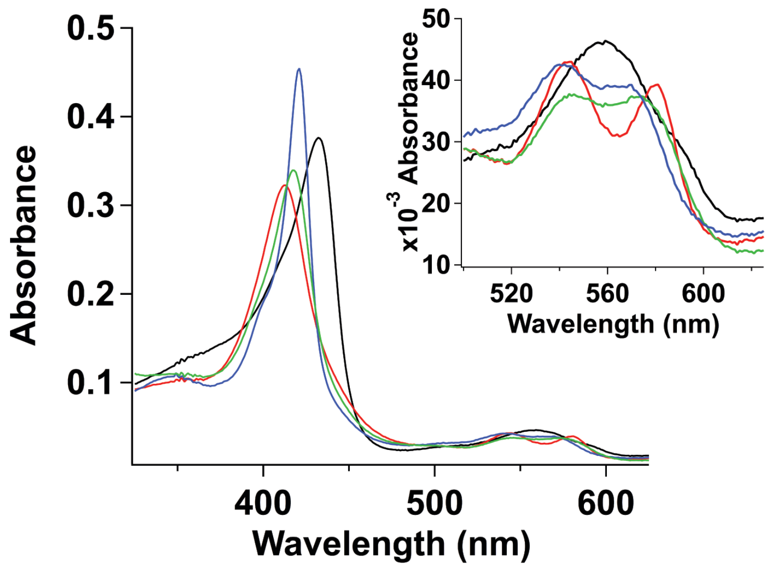

Figure S3. Ultraviolet-visible absorption spectra of *Pcc*GCS H237A/K238A. Red, Fe(II)-O_2; green, Fe(II)-NO; blue, Fe(II)-CO; black, Fe(II) unligated.

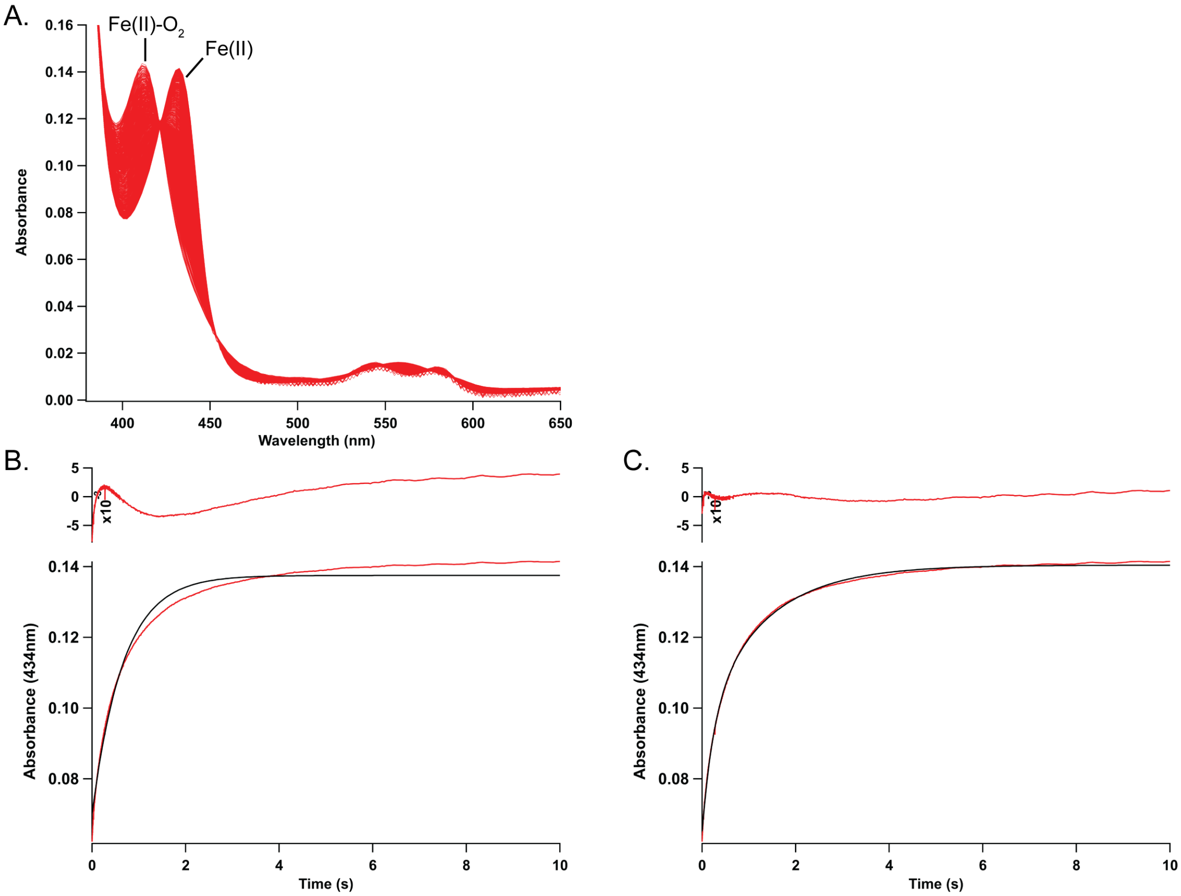

Figure S4. Representative stopped flow spectra and fits from an O_2 dissociation experiment of the π-helix double mutant. A) Representative stopped flow spectra for O2 dissociation from *Pcc*GCS FeII−O_2 H237A/K238A. (B) Raw data (red) and single

exponential fit (black) for *Pcc*GCS FeII−O_2 H237A/K238A. Residuals (difference between actual data and fit) are shown in red above the plot. (C) Raw data (red) and double exponential fit (black) for *Pcc*GCS FeII−O_2 H237A/K238A. Residuals (difference between actual data and fit) are shown in red above the plot.

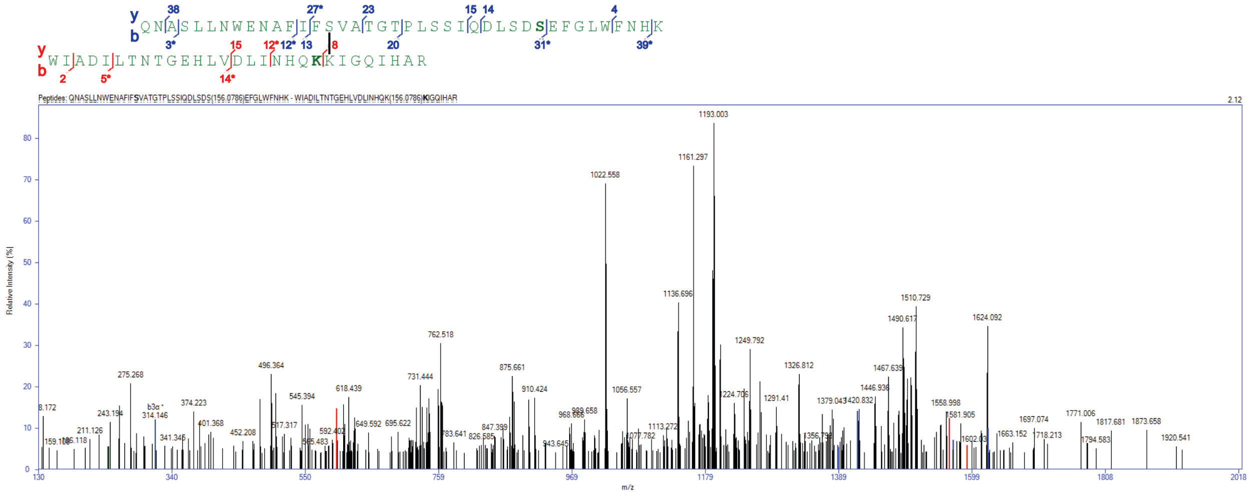

Figure S5. Example of high resolution fragmentation spectra of *Pcc*GCS WT globin domain αF helix crosslinked with middle domain π-helix. Peaks represent fragmented ions that are annotated in red and blue based on peptides involved in crosslink, and y (C-terminal fragment) and b (N-terminal fragment) ions show where peptide backbone was broken. “ * “ indicates fragmented ion peak is found in spectra shown.

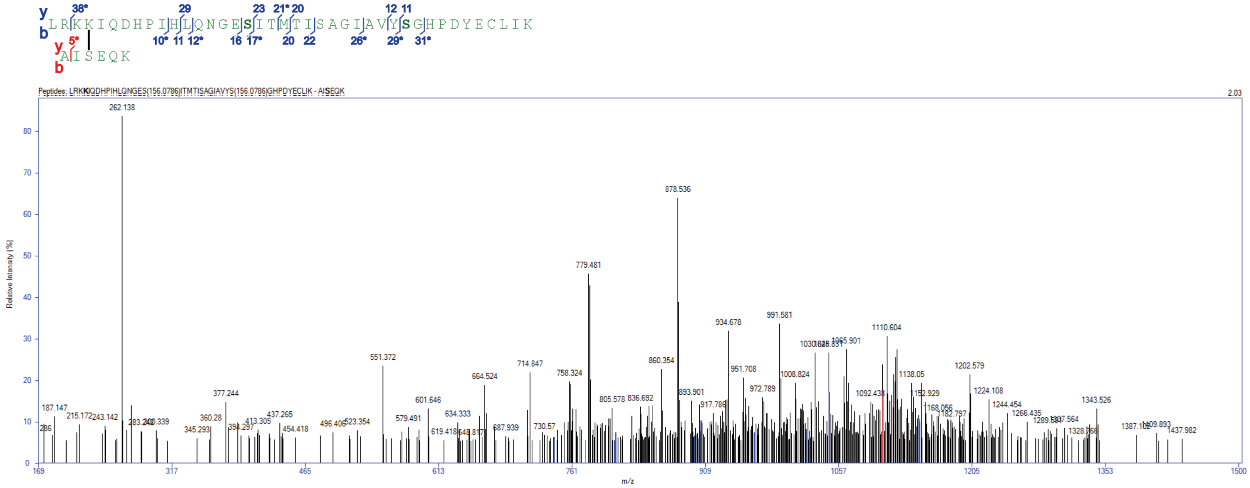

Figure S6. Example of high resolution fragmentation spectra of *Pcc*GCS WT globin domain helix αB crosslinked with DGC domain helix αD. Peaks represent fragmented ions that are annotated in red and blue based on peptides involved in crosslink, and y (C-terminal fragment) and b (N-terminal fragment) ions show where peptide backbone was broken. “ * “ indicates fragmented ion peak is found in spectra shown.

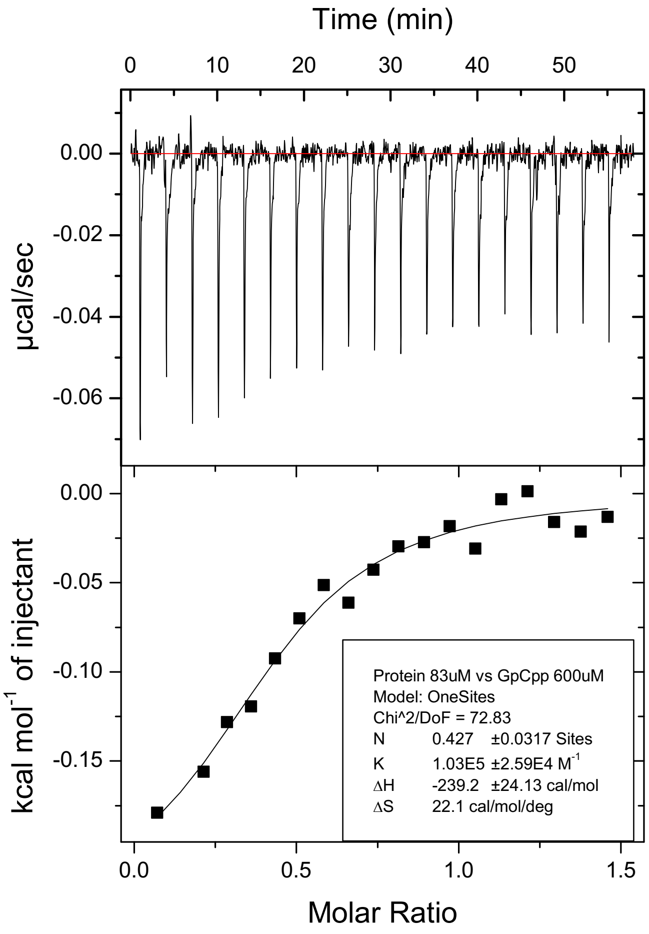

Figure S7. ITC titration of *Pcc*GCS H237A/K238A binding to guanosine-5'-(α-thio)-triphosphate, sodium salt (GpCpp). Binding is reported as K_a = 1.03 x 10^5 M^{-1}; the K_d (calculated as the inverse of K_a) = 9.7 μM.

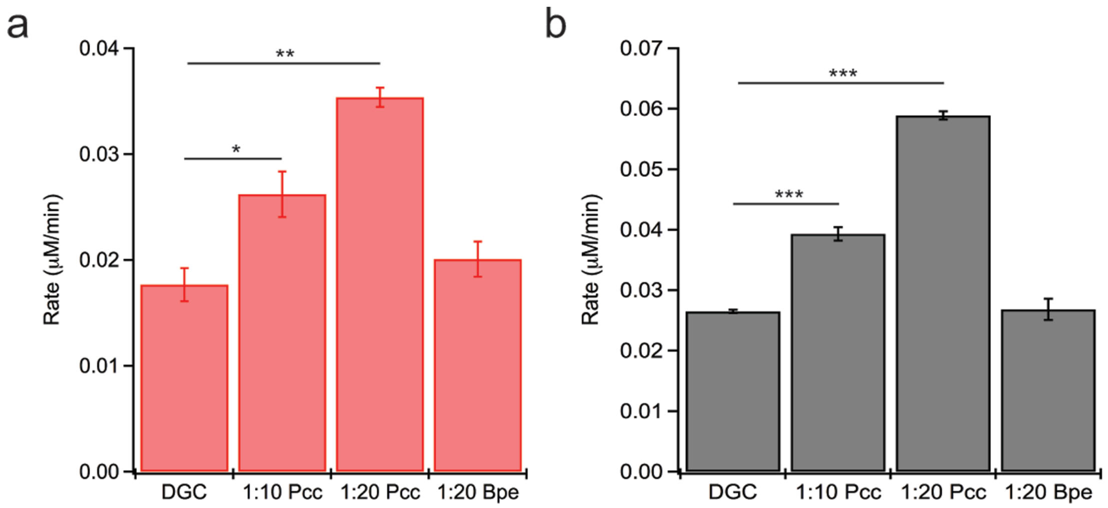

Figure S8. Isolated diguanylate cyclase activity in the presence of isolated globin domains. (**a**) Aerobic kinetic rates of MBP-*Pcc*DGC with and without isolated *Pcc*Globin and *Bpe*Globin. (**b**) Anaerobic kinetic rates of MBP-*Pcc*DGC with and without isolated *Pcc*Globin and *Bpe*Globin. DGC – 2.83/3.18 μM MBP-*Pcc*DGC (Fe(II)-O_2 and Fe(II), respectively) / no globin; 1:10 Pcc - 2.83/3.18 μM MBP-*Pcc*DGC (Fe(II)-O_2 and Fe(II), respectively) / 30 μM *Pcc*Globin; 1:20 Pcc - 2.83/3.18 μM MBP-*Pcc*DGC (Fe(II)-O_2 and Fe(II), respectively) / 60 μM *Pcc*Globin; 1:20 Bpe - 2.83/3.18 μM MBP-*Pcc*DGC (Fe(II)-O_2 and Fe(II), respectively) / 60 μM *Bpe*Globin. Reactions were performed in triplicate. Error bars represent standard deviations (*, $P = 0.0052$; **, $P = 0.0001$; and ***, $P < 0.0001$).
